# Changes in Drug List Prices and Amounts Paid by Patients and Insurers

**DOI:** 10.1001/jamanetworkopen.2020.28510

**Published:** 2020-12-09

**Authors:** Eric J. Yang, Emilio Galan, Robert Thombley, Andrew Lin, Jaeyun Seo, Chien-Wen Tseng, Jack S. Resneck, Peter B. Bach, R. Adams Dudley

**Affiliations:** 1 Department of Dermatology, Warren Alpert Medical School, Brown University, Providence, Rhode Island; 2 Center for Healthcare Value, Philip R. Lee Institute for Health Policy Studies, University of California, San Francisco; 3 Department of Family Medicine and Community Health, University of Hawaii John A. Burns School of Medicine, Honolulu; 4 Department of Dermatology, University of California, San Francisco; 5 Center for Health Policy and Outcomes, Memorial Sloan Kettering Cancer Center, New York, New York; 6 School of Medicine, School of Public Health, Institute for Health Informatics, University of Minnesota, Minneapolis; 7 Minneapolis VA Medical Center, Minneapolis, Minnesota

## Abstract

**Importance:**

High out-of-pocket drug costs can cause patients to skip treatment and worsen outcomes, and high insurer drug payments could increase premiums. Drug wholesale list prices have doubled in recent years. However, because of manufacturer discounts and rebates, the extent to which increases in wholesale list prices are associated with amounts paid by patients and insurers is poorly characterized.

**Objective:**

To determine whether increases in wholesale list prices are associated with increases in amounts paid by patients and insurers for branded medications.

**Design, Setting, and Participants:**

Cross-sectional retrospective study analyzing pharmacy claims for patients younger than 65 years in the IBM MarketScan Commercial Database and pricing data from SSR Health, LLC, between January 1, 2010, and December 31, 2016. Pharmacy claims analyzed represent claims of employees and dependents participating in employer health benefit programs belonging to large employers. Rebate data were estimated from sales data from publicly traded companies. Analysis focused on the top 5 patent-protected specialty and 9 traditional brand-name medications with the highest total drug expenditures by commercial insurers nationwide in 2014. Data were analyzed from July 2017 to July 2020.

**Exposures:**

Calendar year.

**Main Outcomes and Measures:**

Changes in inflation-adjusted amounts paid by patients and insurers for branded medications.

**Results:**

In this analysis of 14.4 million pharmacy claims made by 1.8 million patients from 2010-2016, median drug wholesale list price increased by 129% (interquartile range [IQR], 78%-133%), while median insurance payments increased by 64% (IQR, 28%-120%) and out-of-pocket costs increased by 53% (IQR, 42%-82%). The mean percentage of wholesale list price accounted for by discounts increased from 17% in 2010 to 21% in 2016, and the mean percentage of wholesale list price accounted for by rebates increased from 22% in 2010 to 24% in 2016. For specialty medications, median patient out-of-pocket costs increased by 85% (IQR, 73%-88%) from 2010 to 2016 after adjustment for inflation and 42% (IQR, 25%-53%) for nonspecialty medications. During that same period, insurer payments increased by 116% for specialty medications (IQR, 100%-127%) and 28% for nonspecialty medications (IQR, 5%-34%).

**Conclusions and Relevance:**

This study’s findings suggest that drug list prices more than doubled over a 7-year study period. Despite rising manufacturer discounts and rebates, these price increases were associated with large increases in patient out-of-pocket costs and insurer payments.

## Key Points

**Question:**

How are increases in wholesale list prices for medications distributed among increases in patient out-of-pocket costs, insurer payments, and changes in rebates and discounts?

**Findings:**

In this cross-sectional analysis of 14.4 million pharmacy claims made by 1.8 million patients for the top 5 patent-protected specialty and 9 traditional brand-name medications with the highest total drug expenditures by commercial insurers in 2014, the median drug wholesale list price (as defined by Average Wholesale Price) increased by 129% from 2010-2016, while median patient out-of-pocket costs increased by 53% and median insurance payments after rebates and discounts increased by 64%.

**Meaning:**

This study’s findings suggest that, after adjusting for inflation, increases in drug list prices are associated with increased patient out-of-pocket costs, which may have implications for cost-related nonadherence, and insurer payments.

## Teaser

This cross-sectional study uses data from the IBM MarketScan Commercial Database to examine the association between increases in wholesale list prices of branded medications and amounts paid by patients and insurers for the same drugs.

## Introduction

Large increases in drug prices outpacing increases in income or general inflation in recent years have put the pharmaceutical industry under scrutiny.^1,2,3,4,5^ As cost-related drug nonadherence is already widespread, rising drug prices raise concerns that patients will be unable to afford their prescriptions, leading to negative health outcomes.^6^

Newly developed medications are often expected to be expensive because of high research and development costs, although there is debate about how to estimate these costs.^7^ Recent reports of substantial increases in list prices of drugs already on the market cannot be explained by development costs and have received considerable media attention^3,4,8,9^ and criticism from lawmakers, insurers, and consumers alike.^1,10,11,12^ However, pharmaceutical industry leaders argue that changes in wholesale list prices do not necessarily lead to large changes in amounts patients or insurers pay for drugs.^13,14,15^ Drug manufacturers have argued that increases in list prices are due to increases in discounts or rebates, not increases in actual amounts paid by patients and payers.^16^

Hernandez et al^17^ recently demonstrated that discounts and rebates have increased, as manufacturers report. However, after accounting for these price reductions, total payments manufacturers received rose faster than general inflation. What remains unknown, however, is how this mix of increases in list prices with rising discounts and rebates affects patients’ out-of-pocket costs. If discounts and rebates are large enough and targeted to patients, patient out-of-pocket costs may not rise as list prices increase. However, this hypothesis cannot be evaluated without evaluating discounts, rebates, and amounts paid by payers and by patients out of pocket in a single analysis. This issue is particularly relevant to clinical practice in which news about list price changes must be translated into discussions about drug costs with patients.

To address these issues, we studied a national sample of employer and commercial insurer pharmaceutical claims and sales data from 2010 to 2016 for drugs previously identified as contributing the most to total drug expenditures for commercial insurers.^18^ We analyzed changes over time in wholesale list prices and amounts paid by patients and insurers after accounting for discounts and rebates to understand how rising list prices are associated with patients’ out-of-pocket costs and the net cost of these drugs to insurers.

## Methods

### Drugs Studied

We focused our analyses on the top 5 patent-protected specialty and 9 traditional brand-name medications with the highest total drug expenditures by commercial insurers nationwide in 2014.^18,19^ The most widely used specialty drugs include treatments for rheumatoid arthritis, Crohn disease, psoriasis, cancer, and HIV, among other conditions.^20^ Specialty medications commonly have high prices and have been implicated in driving rising national drug expenditures.^18,21^

### Database and Study Population

We used Health Insurance Portability and Accountability Act–compliant, deidentified, patient-level outpatient pharmacy claims data from the IBM MarketScan Commercial Database. This database represents the claims of 67.4 million employees and dependents participating in employer health benefit programs belonging to large employers between January 1, 2010, and December 31, 2016. The plans represented include a variety of fee-for-service, preferred provider organization, and capitated health plans. For clarity, we refer to these collectively as *insurers*. Claims for patients aged 65 years and older were excluded because of likely confounding differences in benefit design secondary to dual coverage with Medicare. We excluded claims in which reported prescribed dose exceeded the maximum clinical dose. This study was determined to be exempt from review by the institutional review board at the University of California, San Francisco because the patient claims data were deidentified. This study followed the Strengthening the Reporting of Observational Studies in Epidemiology (STROBE) reporting guideline for cross-sectional studies.

### Average Wholesale Price (List Price), Discounts, and Out-of-Pocket Costs

Each pharmacy claim reports the Average Wholesale Price (AWP) for the drug prescribed, actual payment to the pharmacy (ie, the discounted price paid by the insurer but not accounting for rebates), and the patient’s out-of-pocket cost for the medication. Because drug manufacturers report substantial discounts and rebates, for this analysis we use the AWP, equivalent to the manufacturer’s suggested retail price, as the starting wholesale list price. Another price available on claims is the Wholesale Acquisition Cost, but manufacturers would argue that this price already includes a discount they offer, so we start with the higher AWP as the wholesale list price. We calculated the manufacturer’s discount for each claim as AWP minus actual payment to the pharmacy.

We converted all costs for each claim into unit price (ie, the cost of each claim was divided by the number of doses listed in that claim), to ensure that variation in prices was not due to differences in dosing. Mean AWP, patient out-of-pocket costs, and discounted price were calculated by totaling all claims for a drug and dividing by the number of claims. Discounts were calculated as the difference between AWP and the discounted price. Mean AWP per unit and discounts were calculated for each drug per quarter, whereas mean patient costs were calculated annually to account for patient deductibles.

### Estimated Rebates

Like Hernandez et al,^17^ we obtained rebate data from SSR Health, LLC, a health care–focused investment research firm, and Symphony Health. SSR Health compiles data from pharmaceutical company investor reports to determine total revenue after all price reductions (including discounts and rebates) have been factored in. This is divided by the total number of prescriptions, estimated by Symphony Health, to calculate the net payment for drugs, accounting for discounts and rebates.

SSR Health estimated discounts to Medicaid payers by using the sum of the Medicaid statutory rebate (23.1% of average manufacturer price) and the pricing penalty for price increases exceeding the Consumer Price Index increase. These are discounts offered to state Medicaid programs as part of “best price” regulations to make drug spending more affordable for Medicaid. The number of units of each medication sold to Medicaid was obtained by SSR Health from the Centers for Medicare & Medicaid Services’ drug utilization files. SSR Health then estimated total revenue from Medicaid by multiplying the discounted Medicaid price by the number of units sold to Medicaid. SSR Health calculated revenue from non-Medicaid sales for each drug by subtracting Medicaid revenue from total revenue. Similarly, the number of non-Medicaid units sold for each medication was estimated by subtracting Medicaid units sold from total units sold. SSR Health then divided non-Medicaid revenue by the number of non-Medicaid units sold to estimate the net price for non-Medicaid insurers. To allow for comparison with the pharmacy claims data (which does not include Medicaid payers), we used only the non-Medicaid sales data from this data set.

We then calculated the mean rebate for non-Medicaid payers as the difference between the mean discounted price paid on claims and the non-Medicaid net price from SSR Health. With these figures, we could assess how the AWP for each drug could be broken down into discounts shown on the claim, rebates passed on to insurers, patient out-of-pocket costs, and insurer payments net of discounts and rebates such that

AWP = (Discount + Rebate) + Insurer payments + Patient Out-of-Pocket Costs.

### Independent and Dependent Variables Assessed

Our primary independent variable was percentage of change in AWP from 2010 to 2016 for medications maintaining patent protection during this period. Our secondary independent variables were percentage of change in AWP from 2010 to 2014 and from 2010 to 2015 for medications maintaining patent protection during these respective periods. Our primary dependent variables were the percentage of change in patient out-of-pocket costs and insurer payments after rebates. Secondary dependent variables included the mean percentage of change in discounts and rebates over the study period. In addition, differences in the changes in AWP, net payments (sum of insurer payments and patient out-of-pocket costs), insurer payments, and patient out-of-pocket costs between specialty medications and nonspecialty medications were evaluated. The proportion of change in AWP accounted for by changes in discounts, rebates passed on to insurers, insurer payments, and patient out-of-pocket costs was computed for all medications.

### Statistical Analysis

We assessed differences in price trends between specialty and nonspecialty medications by comparing the proportion of increase in AWP from 2010 to 2016 accounted for by changes in rebates, discounts, insurer payments, and patient out-of-pocket costs between these 2 groups of drugs. Median values were used as a measure of central tendency to reduce the impact of outlier claims or benefits designs. All prices and payments shown were adjusted to 2016 dollars using the Consumer Price Index for All Urban Consumers. Analyses were performed with Excel 2013, version 15.0.4535.1507 (Microsoft Corp). Data were analyzed from July 2017 to July 2020.

### Sensitivity Analysis

Some information about Medicaid discounts and rebates, such as best price discounts or rebates required by individual states, was not available to SSR Health. However, we could calculate a range of possible Medicaid prices. Because total discount and rebate amounts given by drug manufacturers are split between Medicaid and non-Medicaid purchasers, assuming higher Medicaid discounts and rebates would result in an estimation of lower non-Medicaid discounts and rebates.

For our main analysis, the estimates made by SSR Health about Medicaid statutory rebates and pricing penalties led to the highest possible estimates of discounts and rebates to the commercial insurers being studied to maximally reflect manufacturer claims about high discount and rebate rates. As a sensitivity analysis and to estimate a range of patient out-of-pocket costs and insurer payments, we also calculated those outcomes with the assumption of every Medicaid unit in the SSR Health data set being rebated at 100% of average manufacturer price. Because this would be the maximum possible discount paid to Medicaid, it would consequently result in the lowest possible discounts and rebates to commercial insurers.

## Results

For 2010-2016, the MarketScan database included data for 67.4 million enrollees who filled at least 1 prescription, with 1.8 million enrollees having 14.4 million pharmacy claims for the drugs of interest (eTable 1 in the Supplement). Taken together, claims for the 14 drugs accounted for $13 billion in spending, or 6.6% of total drug spending during this study period. The drugs included in our analysis consisted of 5 specialty drugs and 9 nonspecialty drugs.

**Figure 1.**
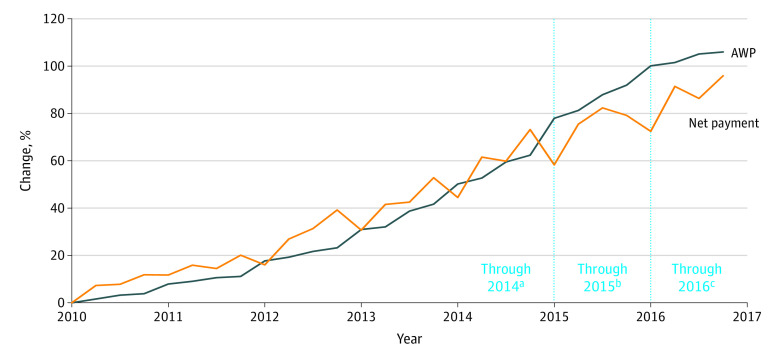
Average Cumulative Percentage of Change in Average Wholesale Price (AWP) and Total Payments per Quarter for Drugs Keeping Patent Protection From 2010 to 2016 All results were adjusted to 2016 dollars using the Consumer Price Index. The AWP represents the total payment, that is, the amount paid by insurance payers and patients after discounts and rebates. ^a^Fourteen drugs kept patent protection through 2014 and were included in analysis from 2010 to 2014. ^b^Eleven drugs kept patent protection through 2015 and were included in analysis from 2014 to 2015. ^c^Nine drugs kept patent protection through 2016 and were included in analysis from 2015 to 2016.

Not all drugs in our study retained patent protection through 2016. Figure 1 shows increases in AWP and net payments (the sum of patient and insurer payments after discounts and rebates) from 2010-2016. For the 14 drugs retaining patent protection through 2014, the median increase in AWP was 59% (interquartile range [IQR], 47%-71%), whereas median net payments increased by 78% (IQR, 27%-111%) above the rate of increase in the Consumer Price Index (eFigure 1 in the Supplement). For the 9 drugs retaining patent protection through 2016, the median increase in AWP was 129% (IQR, 78%-133%) from 2010 to 2016, whereas median net payments increased by 74% (IQR, 27%-158%; eFigure 1 in the Supplement).

From 2010 to 2016, the median out-of-pocket patient payments and insurance payments increased by 53% (IQR, 42%-82%) and 64% (IQR, 28%-120%), respectively. During this time, manufacturer discounts and rebates passed on to insurers rose by a median of 163% (IQR, 130%-212%) and 103% (IQR, −18% to 293%). As a result, the mean percentage of wholesale list price accounted for by discounts increased from 17% in 2010 to 21% in 2016, and the mean percentage of wholesale list price accounted for by rebates increased from 22% in 2010 to 24% in 2016 (eFigure 2 in the Supplement).

The amounts paid by patients out of pocket and by insurers increased for most drugs included in our analysis from 2010 to 2016 (Table 1). For Humalog and Nexium, insurer expenditures decreased slightly.

**Table 1.** Annual Change in Average Wholesale Price (AWP) and Cost by Patients and Insurers After Rebates per Unit of Medication, Ordered by Increase in Patient Out-of-Pocket Cost

Medication	Annual cost increase above rate of inflation, %^a^		
	AWP	Insurer	Patient
Specialty			
Stelara	8.6	14.1	6.3
Gleevec	15.4	17.8	7.6
Atripla	6.2	8.5	10.5
Enbrel	13.6	16.2	11.0
Humira	14.3	13.3	11.3
Nonspecialty			
Crestor	10.5	4.4	3.2
Januvia	10.3	0.8	3.5
Lantus	15.9	5.0	3.8
Abilify	12.7	13.4	4.5
Androgel	10.1	23.4	4.6
Nexium	7.6	−4.9	4.6
Lyrica	14.5	17.0	6.0
Humalog	13.8	−1.9	7.3
Vyvanse	9.6	4.3	8.6

^a^ The reported change per year is net of the rate of general inflation reported from 2010 to end of patent protection or 2016, whichever came first. All results have been adjusted to 2016 dollars using the Consumer Price Index.

Among all study drugs that retained patent protection from 2010 to 2014, every $1 increase in AWP was associated with a median increase in patient out-of-pocket costs of $0.04 (range, $0.01-$0.19) (Table 2). Patient out-of-pocket costs increased $0.04 (range, $0.03-$0.19) for nonspecialty drugs and $0.03 (range, $0.01-$0.04) for specialty drugs. Insurer payments for this period increased $0.63 (range, −$0.07 to $1.61) for all eligible study drugs during this period. Insurer payments increased $0.13 (range, −$0.07 to $1.61) for nonspecialty medications and $0.81 (range, $0.67-$1.15) for specialty drugs for every $1 increase in AWP during this period.

**Table 2.** Median Absolute Increase in Insurer and Patient Payments per $1 Increase in Average Wholesale Price for Medications Maintaining Patent Protection During Each of the Studied Periods^a^

Type of payment	Median (range), $		
	2010-2014 (14 drugs)	2010-2015 (11 drugs)	2010-2016 (9 drugs)
Insurer payments			
Overall	0.63 (−0.07 to 1.61)	0.56 (0.05 to 0.93)	0.57 (−0.04 to 1.02)
Specialty	0.81 (0.67 to 1.15)	0.75 (0.66 to 0.93)	0.74 (0.57 to 1.02)
Nonspecialty	0.13 (−0.07 to 1.61)	0.12 (0.05 to 0.56)	0.13 (−0.04 to 0.62)
Patient payments			
Overall	0.04 (0.01 to 0.19)	0.04 (0.01 to 0.20)	0.04 (0.01 to 0.20)
Specialty	0.03 (0.01 to 0.04)	0.02 (0.01 to 0.05)	0.03 (0.01 to 0.06)
Nonspecialty	0.04 (0.03 to 0.19)	0.05 (0.02 to 0.20)	0.05 (0.02 to 0.20)

^a^ All results have been adjusted to 2016 dollars using the Consumer Price Index.

Among all study drugs retaining patent protection from 2010 to 2016, every $1 increase in AWP was associated with a median increase in patient out-of-pocket costs of $0.04 (range, $0.01-$0.20). Patient out-of-pocket costs increased $0.05 (range, $0.02-$0.20) for nonspecialty drugs and $0.03 (range, $0.01-$0.06) for specialty drugs. Insurer payments for this period increased $0.57 (range, −$0.04 to $1.02) for all eligible study drugs during this period. Insurer payments increased $0.13 (range, −$0.04 to $0.62) for nonspecialty drugs and $0.74 (range, $0.57-$1.02) for specialty drugs for every $1 increase in AWP during this period.

**Figure 2.**
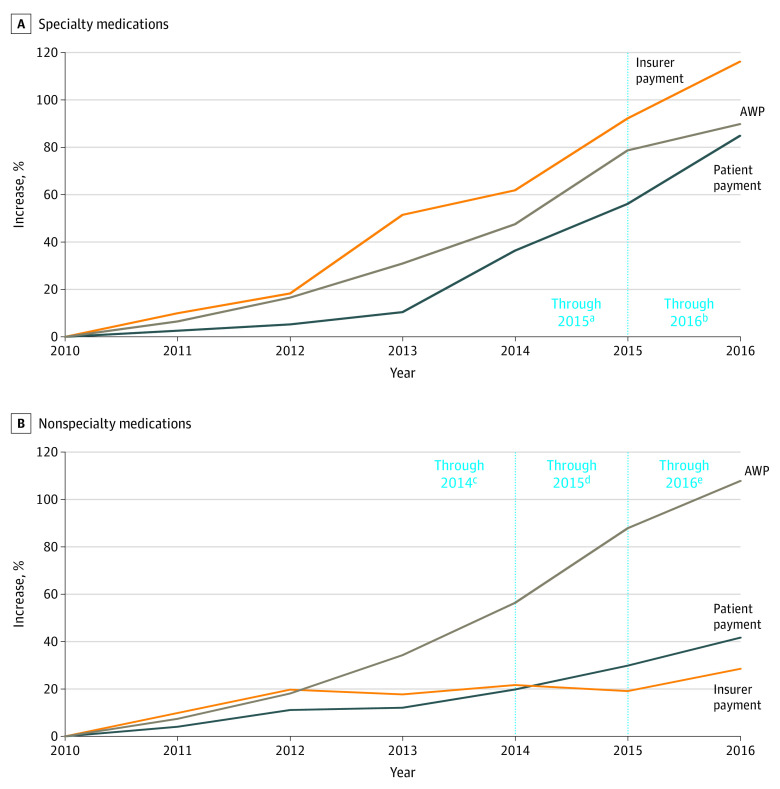
Changes in Median Annual Average Wholesale Price (AWP) and Patient and Payer Expenditures per Medication Relative to 2010 for Specialty and Nonspecialty Medications A, All results were adjusted to 2016 dollars using the Consumer Price Index. B, All results were adjusted to 2016 dollars using the Consumer Price Index. The AWP represents total payment, that is, the amount paid by insurance payers and patients after discounts and rebates. ^a^Five specialty drugs kept patent protection through 2015 and were included in analysis from 2010 to 2015. ^b^Four specialty drugs kept patent protection through 2016 and were included in analysis from 2015 to 2016. ^c^Nine nonspecialty drugs kept patent protection through 2014 and were included in analysis from 2010 to 2014. ^d^Six nonspecialty drugs kept patent protection through 2015 and were included in analysis from 2014 to 2015. ^e^Five nonspecialty drugs kept patent protection through 2016 and were included in analysis from 2015 to 2016.

In total, median patient out-of-pocket costs for specialty medications increased by 85% (IQR, 73%-88%) after adjustment for inflation as compared with 42% (IQR, 25%-53%) for nonspecialty medications from 2010 to 2016, while insurer payments for specialty medications increased by 116% (IQR, 100%-127%) compared with 28% (IQR, 5%-34%) for nonspecialty medications (Figure 2).

In our sensitivity analysis in which we assessed the range of possible discounts and rebates to commercial insurers, patient out-of-pocket costs were unchanged. Median insurer payment increases were slightly lower than those in our primary analysis, although still far above the rate of increase in the Consumer Price Index. With these assumptions, median insurer payments for drugs retaining patent protection through 2016 increased by 61% (IQR, 34%-122%) after adjustment for inflation.

## Discussion

In a large, national sample of patients with commercial insurance, we found that increases in drug wholesale list prices were accompanied by increases in out-of-pocket payments by patients and by insurers. Increases in payments for specialty medications from 2010 to 2016 were greater than those for nonspecialty medications in our analysis over the study period.

Pharmaceutical manufacturers have argued that increases in drug wholesale list prices are accompanied by corresponding large increases in discounts and rebates, reducing the impact of list price changes on patients and insurers.^13,14,15^ Our analysis corroborates that rebates and discounts are large, and the proportion of AWP attributed to discounts and rebates did increase from 2010 to 2016 (eFigure 3 in the Supplement). However, after accounting for these changes, we show that increases in wholesale list prices were associated with increases in patient out-of-pocket costs and insurer payments for the drugs studied. This varies by drug, but wholesale list prices, insurer payments, and patient out-of-pocket costs all increased from 2010 to 2016 at rates above the rate of increase in the Consumer Price Index (eTable 2 in the Supplement).

Although patient out-of-pocket costs for drugs vary greatly, patients paid a median of 53% more for these drugs in 2016 than they did in 2010, after adjusting for general inflation. During the same period, median household income rose 8.6%,^22^ so these out-of-pockets costs have increased relative to income. Because we used claims to determine out-of-pocket costs, this finding persists despite coupons and patient assistance programs aimed at lowering patient spending. This observed rise in patient out-of-pocket costs with increasing drug list prices is of particular concern to clinicians because high drug costs can lead to poor drug regimen adherence, increased use of utilization management systems such as prior authorizations and step therapy, and adverse health outcomes.^6,20^ Although patients paid a smaller percentage of the wholesale list price over time, the absolute amount that they paid for the drugs we studied continued to increase at rates higher than general inflation or the growth of income, which may have important implications for the ever larger population of patients with chronic diseases.^23,24^ This study highlights that increases in prices for drugs that have been on the market for several years increase the cost of treatment for patients.

Spending for specialty drugs has increased significantly over the past decade and is projected to contribute to rapidly increasing health care costs.^20,25,26^ Our results indicate that discounts and rebates for these drugs are lower relative to list prices than discounts and rebates for nonspecialty medications. Insurer payments for Nexium and Humalog did not outpace inflation in our analysis, presumably because they face more competition from similar drugs in their classes. Manufacturers of single-source specialty medications may offer fewer price concessions because most face little market competition.^27^ Consequently, insurer payments for specialty drugs are also rising at a much higher rate than payments for nonspecialty medications, also increasing coinsurance costs. Although patient out-of-pocket costs are a smaller proportion of the total price of specialty medications than nonspecialty medications, specialty drugs are still more costly to patients. As use of these drugs increases, the high costs may affect patients through higher out-of-pocket spending per prescription and rising premiums, as well as both government programs and commercial insurers.^18,28^

### Limitations

Our study has several limitations. Given differences in pricing of generic medications with regard to discounts and rebates, we opted not to include medications that did not have patent protection during our study period. The findings of our study reflect the pricing breakdown of the 14 medications accounting for the greatest portion of insurer medication expenditures and may not be generalizable to other medications. In addition, several of the analyzed medications lost patent protection shortly after the study period. Further study is needed to characterize how impending patent protection expiration affects list prices, discounts, rebates, and amounts paid for medications.

We were also unable to control for changes in cost sharing during this period, which may also contribute to changes in amounts paid for medications. Furthermore, contracting and pricing are different for Medicare, Medicaid, the 340B program, and the Department of Veterans Affairs, so it is not clear how generalizable our findings are to those markets.

In addition, we could not obtain precise estimates of all Medicaid discounts and rebates, resulting in some uncertainty about the total discounts and rebates offered to the commercial insurers we studied. Therefore, our sensitivity analyses provide only a range of possible insurer payments rather than exact figures. However, under any set of assumptions, we found that increases in list prices resulted in increases in insurer payments. This limitation has no effect on our estimates of patient out-of-pocket costs, since these were identified directly from claims data.

Our study also does not characterize who receives manufacturers’ rebates in detail. Rather, we report what can be directly measured: the amount that is passed on to insurers. Insurers may then pass some of these savings on to employers, but rebates are also frequently paid—at least in part—to pharmaceutical benefits managers.^29^ We are not aware of data that allow description of how rebates are shared among these relevant parties, but they are not shared with patients.

Finally, we were not able to measure other mechanisms by which rising drug prices may increase patient out-of-pocket costs, such as increasing insurance premiums. Additional study of the association between rising insurer payments and these other patient out-of-pocket costs is needed to further clarify the total effect of these pricing changes on patients.

## Conclusions

In this cross-sectional study, we found that increases in drug wholesale list prices are associated with increases in net patient out-of-pocket costs and insurer payments. This finding suggests that, although discounts and rebates significantly reduce the amount paid for drugs and have increased over the past several years, they have not prevented an inflation-adjusted rise in patients’ and insurers’ costs. This could have both important clinical implications for patient outcomes and an impact on total health care expenditures.

## Notes

Supplement. **eTable 1.** Characteristics of the Patient Population in the Sample **eFigure 1.** Average Cumulative Percent Change in Average Wholesale Price (AWP) and Net Payments per Quarter **eFigure 2.** Mean Percent of Average Wholesale Price (AWP) Attributed to Discounts and Rebates by Quarter from 2010 to 2016 **eFigure 3.** Proportions of Average Wholesale Price (AWP) Accounted for by Discounts, Net Rebates Passed Onto Insurers, Insurer Expenditures, and Patient Out-of-Pocket Payments in 2010 and 2016 for Non-specialty Medications and Specialty Medications **eTable 2.** Median Annual Percent Increase in List Prices, Price Reductions, and Payments for Medications Maintaining Patent Protection During Each of the Studied Time Periods

## References

[1] Humer C Makers took big price increases on widely used U.S. drugs. Reuters. Updated April 5, 2016. Accessed May 26, 2018. https://www.reuters.com/article/us-usa-healthcare-drugpricing/exclusive-makers-took-big-price-increases-on-widely-used-u-s-drugs-idUSKCN0X10TH

[2] Pollack A Big price increase for tuberculosis drug is rescinded. *New York Times* Updated September 21, 2015. Accessed May 27, 2018. https://www.nytimes.com/2015/09/22/business/big-price-increase-for-tb-drug-is-rescinded.html

[3] Pollack A Drug goes from $13.50 a tablet to $750, overnight. *New York Times* Updated September 20, 2015. Accessed May 27, 2018. https://www.nytimes.com/2015/09/21/business/a-huge-overnight-increase-in-a-drugs-price-raises-protests.html

[4] Rubin R EpiPen price hike comes under scrutiny. Lancet. 2016;388(10051):1266. doi:10.1016/S0140-6736(16)31708-1 27673458

[5] Hernandez I, Good CB, Cutler DM, Gellad WF, Parekh N, Shrank WH The contribution of new product entry versus existing product inflation in the rising costs of drugs. Health Aff (Millwood). 2019;38(1):76-83. doi:10.1377/hlthaff.2018.05147 30615532

[6] Shrank WH, Hoang T, Ettner SL, The implications of choice: prescribing generic or preferred pharmaceuticals improves medication adherence for chronic conditions. Arch Intern Med. 2006;166(3):332-337. doi:10.1001/archinte.166.3.332 16476874

[7] Avorn J The $2.6 billion pill–methodologic and policy considerations. N Engl J Med. 2015;372(20):1877-1879. doi:10.1056/NEJMp1500848 25970049

[8] Egan M. Painkiller that once cost $138 is now $2,979. *CNN Business* Published February 15, 2018. Accessed May 26, 2018. https://money.cnn.com/2018/02/15/investing/drug-prices-vimovo-horizon-painkiller/index.html

[9] Walker J For prescription drug makers, price increases drive revenue. *Wall Street Journal* Accessed May 26, 2018. https://www.wsj.com/articles/for-prescription-drug-makers-price-increases-drive-revenue-1444096750

[10] McCarthy M US Senate committee launches investigation into drug pricing. BMJ. 2015;351:h5989. doi:10.1136/bmj.h5989 26553078

[11] Bach PB Why drugs cost so much. *New York Times* Updated January 14, 2015. Accessed May 26, 2018. https://www.nytimes.com/2015/01/15/opinion/why-drugs-cost-so-much.html

[12] Houston AR, Beall RF, Attaran A Upstream solutions for price-gouging on critical generic medicines. J Pharm Policy Pract. 2016;9:15. doi:10.1186/s40545-016-0064-8 27141308 PMC4852412

[13] Transforming lives, advancing hope: 2019 Jansen U.S. transparency report. Janssen Pharmaceutical Companies of Johnson & Johnson. Updated 2018-03-02. Accessed May 26, 2018. http://jnj-janssen.brightspotcms.com/us/us-pharmaceutical-transparency-report/pricing-and-patient-access

[14] Testimony of Mylan CEO Heather Bresch before the United States House of Representatives Committee on Oversight and Government Reform. Accessed November 4, 2020. https://www.mylan.com/-/media/mylancom/files/news/oral-testimony-of-mylan-ceo-heather-bresch-before-the-united-states-house-of-representatives-committee-on-oversight-and-government-reform.pdf?la=en

[15] Tirell M Johnson & Johnson lifts lid on drug pricing data, shows 3.5% net hike in 2016 *CNBC* Published February 27, 2017. Updated February 27, 2017. Accessed May 26, 2018. https://www.cnbc.com/2017/02/27/johnson-johnson-lifts-lid-on-drug-pricing-data-shows-35-net-hike-in-2016.html

[16] Pharmaceutical Research and Manufacturers of America. Let's talk about cost. Published 2018. Accessed March 4, 2020. https://www.letstalkaboutcost.org/

[17] Hernandez I, San-Juan-Rodriguez A, Good CB, Gellad WF Changes in list prices, net prices, and discounts for branded drugs in the US, 2007-2018. JAMA. 2020;323(9):854-862. doi:10.1001/jama.2020.1012 32125403 PMC7054846

[18] Dusetzina SB Share of specialty drugs in commercial plans nearly quadrupled, 2003-14. Health Aff (Millwood). 2016;35(7):1241-1246. doi:10.1377/hlthaff.2015.1657 27385240

[19] Comprehensive Specialty Pharmacy Drug List. CVS Specialty. Published October 2019. Accessed September 20, 2020. https://www.caremark.com/portal/asset/IBM_Specialty_Drug_List.pdf

[20] Lotvin AM, Shrank WH, Singh SC, Falit BP, Brennan TA Specialty medications: traditional and novel tools can address rising spending on these costly drugs. Health Aff (Millwood). 2014;33(10):1736-1744. doi:10.1377/hlthaff.2014.0511 25288417

[21] Cuckler GA, Sisko AM, Poisal JA, National health expenditure projections, 2017-26: despite uncertainty, fundamentals primarily drive spending growth. Health Aff (Millwood). 2018;37(3):482-492. doi:10.1377/hlthaff.2017.1655 29443634

[22] US Census Bureau. Real median household income in the United States [MEHOINUSA672N], retrieved from FRED, Federal Reserve Bank of St Louis. Updated September 16, 2020. Accessed September 20, 2020 https://fred.stlouisfed.org/series/MEHOINUSA672N

[23] Ward BW, Schiller JS, Goodman RA Multiple chronic conditions among US adults: a 2012 update. Prev Chronic Dis. 2014;11:E62. doi:10.5888/pcd11.130389 24742395 PMC3992293

[24] Piette JD, Heisler M, Wagner TH Cost-related medication underuse among chronically ill adults: the treatments people forgo, how often, and who is at risk. Am J Public Health. 2004;94(10):1782-1787. doi:10.2105/AJPH.94.10.1782 15451750 PMC1448534

[25] Martin AB, Hartman M, Washington B, Catlin A; National Health Expenditure Accounts Team National health spending: faster growth in 2015 as coverage expands and utilization increases. Health Aff (Millwood). 2017;36(1):166-176. doi:10.1377/hlthaff.2016.1330 27913569

[26] Kesselheim AS, Avorn J, Sarpatwari A The high cost of prescription drugs in the United States: origins and prospects for reform. JAMA. 2016;316(8):858-871. doi:10.1001/jama.2016.11237 27552619

[27] Dusetzina SB, Bach PB Prescription drugs-list price, net price, and the rebate caught in the middle. JAMA. 2019;321(16):1563-1564. doi:10.1001/jama.2019.2445 30840047

[28] Schumock GT, Li EC, Wiest MD, National trends in prescription drug expenditures and projections for 2017. Am J Health Syst Pharm. 2017;74(15):1158-1173. doi:10.2146/ajhp170164 28533252

[29] Dusetzina SB, Conti RM, Yu NL, Bach PB Association of prescription drug price rebates in Medicare Part D with patient out-of-pocket and federal spending. JAMA Intern Med. 2017;177(8):1185-1188. doi:10.1001/jamainternmed.2017.1885 28558108 PMC5722464

