# Supplementary material for: Changes in Drug List Prices and Amounts Paid by Patients and Insurers
Source: JAMA Netw Open. 2020 Dec 9;3(12):e2028510. doi: 10.1001/jamanetworkopen.2020.28510 (PMC7726630; doi:10.1001/jamanetworkopen.2020.28510)
Supplement: Supplement. — eTable 1. Characteristics of the Patient Population in the Sample eFigure 1. Average Cumulative Percent Change in Average Wholesale Price (AWP) and Net Payments per Quarter eFigure 2. Mean Percent of Average Wholesale Price (AWP) Attributed to Discounts and Rebates by Quarter from 2010 to 2016 eFigure 3. Proportions of Average Wholesale Price (AWP) Accounted for by Discounts, Net Rebates Passed Onto Insurers, Insurer Expenditures, and Patient Out-of-Pocket Payments in 2010 and 2016 for Non-specialty Medications and Specialty Medications eTable 2. Median Annual Percent Increase in List Prices, Price Reductions, and Payments for Medications Maintaining Patent Protection During Each of the Studied Time Periods [file jamanetwopen-e2028510-s001.pdf]

Supplementary Online Content

Yang EJ, Galan E, Thombley R, et al. Changes in drug list prices and amounts paid by patients and insurers. *JAMA Netw Open.* 2020;3(12): e2028510. doi:10.1001/jamanetworkopen.2020.28510

eTable 1. Characteristics of the Patient Population in the Sample

eFigure 1. Average Cumulative Percent Change in Average Wholesale Price (AWP) and Net Payments per Quarter

eFigure 2. Mean Percent of Average Wholesale Price (AWP) Attributed to Discounts and Rebates by Quarter from 2010 to 2016

eFigure 3. Proportions of Average Wholesale Price (AWP) Accounted for by Discounts, Net Rebates Passed Onto Insurers, Insurer Expenditures, and Patient Out-of-Pocket Payments in 2010 and 2016 for Non-specialty Medications and Specialty Medications

eTable 2. Median Annual Percent Increase in List Prices, Price Reductions, and Payments for Medications Maintaining Patent Protection During Each of the Studied Time Periods

This supplementary material has been provided by the authors to give readers additional information about their work.

©2020 Yang EJ et al. *JAMA Network Open*

eTable 1. Characteristics of the patient population in the sample

Characteristic	Value
Total population	1,849,513
Total number of prescriptions	14,392,108
Age	
0-17	10.9%
18-34	13.4%
35-44	14.2%
45-54	25.8%
55-64	35.8%
Sex	
Male	53.0%
Female	47.0%
Employee Status	
Active Full Time	51.9%
Active Part time	0.9%
Early Retiree	7.5%
Medicare Eligible Retiree	1.1%
Retiree (unknown status)	1.1%
COBRA continuee	1.2%
Long Term Disability	0.4%
Surviving Spouse	0.3%
Other/Unknown	35.5%
Region	
Northeast	14.8%
North Central	22.9%
South	50.2%
West	12.1%
Prescription Claim Characteristics	
Prescription Type	
Initial	56.3%
Refill	43.7%
Prescription Source	
Retail	80.3%
Mail order	19.1%
Unknown	0.6%

©2020 Yang EJ et al. *JAMA Network Open*

eFigure 1. Average cumulative percent change in Average Wholesale Price (AWP) and net payments per quarter.
All results were adjusted to 2016 dollars using the Consumer Price Index (CPI).

- Panel A: for 14 drugs which kept patent protection from 2010 through 2014.
- Panel B: for 11 drugs which kept patent protection from 2010 through 2015.
- Panel C: for 9 drugs which kept patent protection from 2010 through 2016.

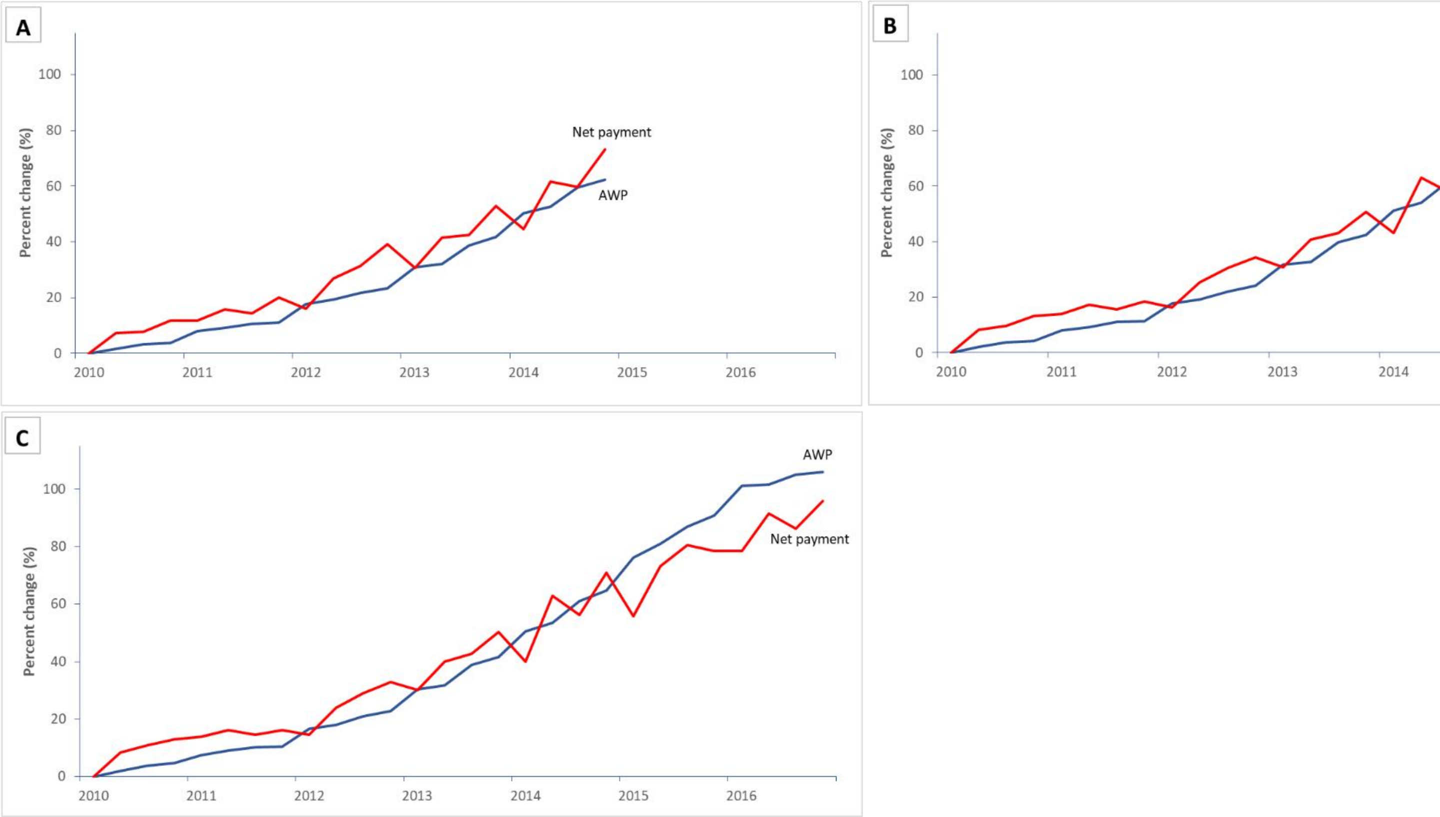

AWP = Average Wholesale Price; Net payment = net amount paid by insurance payers and patients after discounts and rebates

©2020 Yang EJ et al. *JAMA Network Open*

eFigure 2. Mean percent of Average Wholesale Price (AWP) attributed to discounts and rebates by quarter from 2010 to 2016.

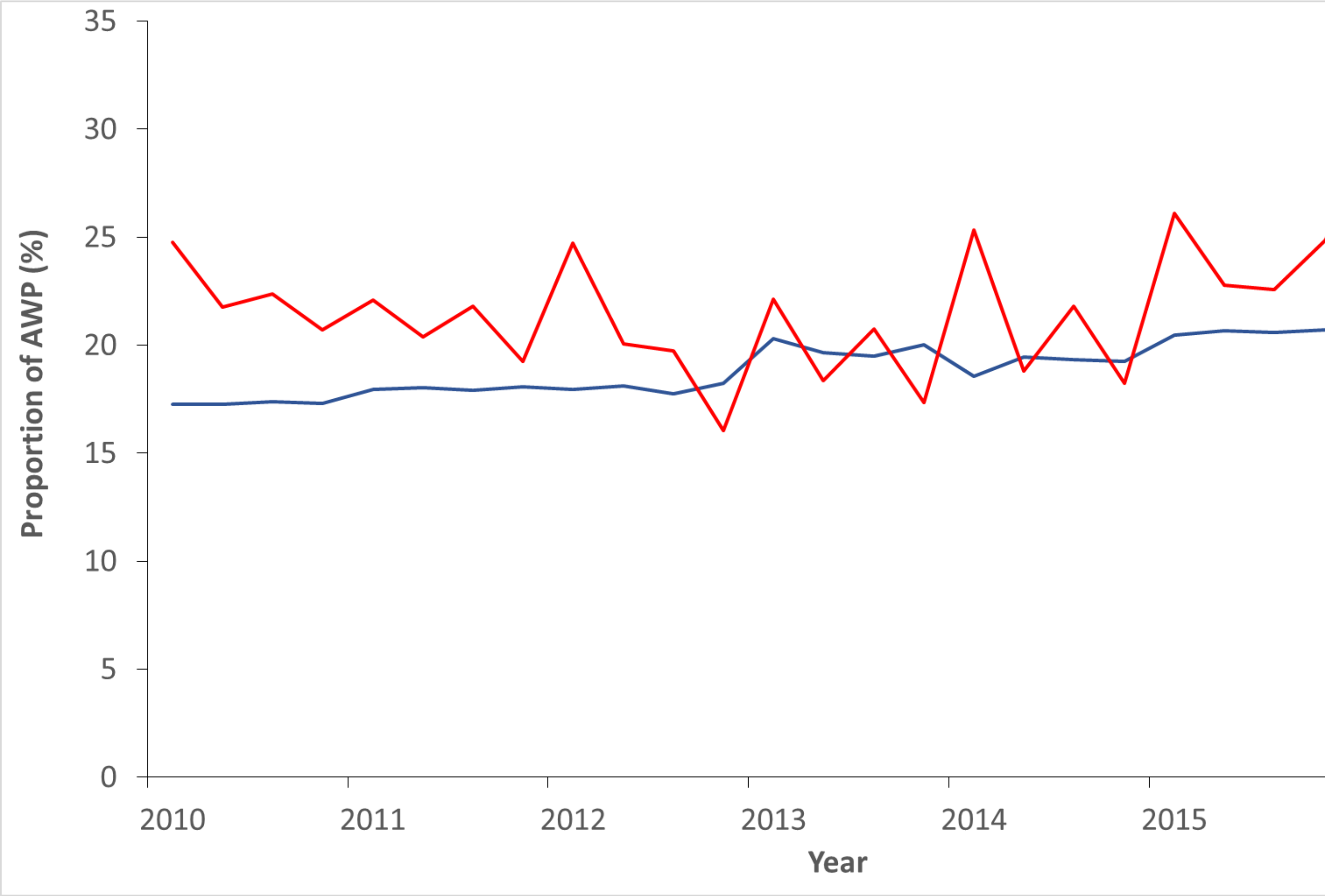

AWP = Average Wholesale Price

All results were adjusted to 2016 dollars using the Consumer Price Index (CPI).

©2020 Yang EJ et al. *JAMA Network Open*

eFigure 3. Proportions of Average Wholesale Price (AWP) accounted for by discounts, net rebates passed onto insurers, insurer expenditures, and patient out-of-pocket payments in 2010 and 2016 for A) non-specialty medications and B) specialty medications

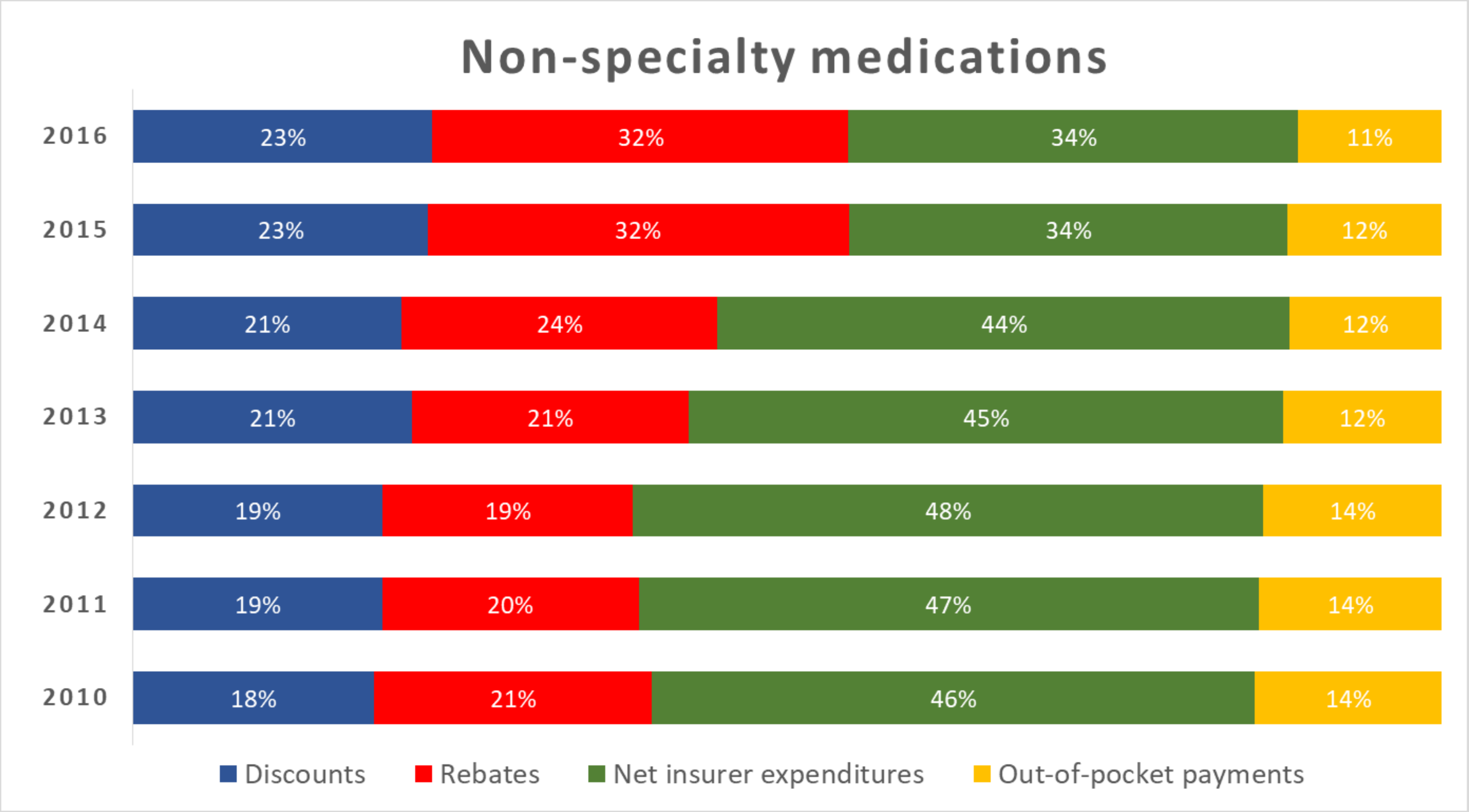

©2020 Yang EJ et al. *JAMA Network Open*

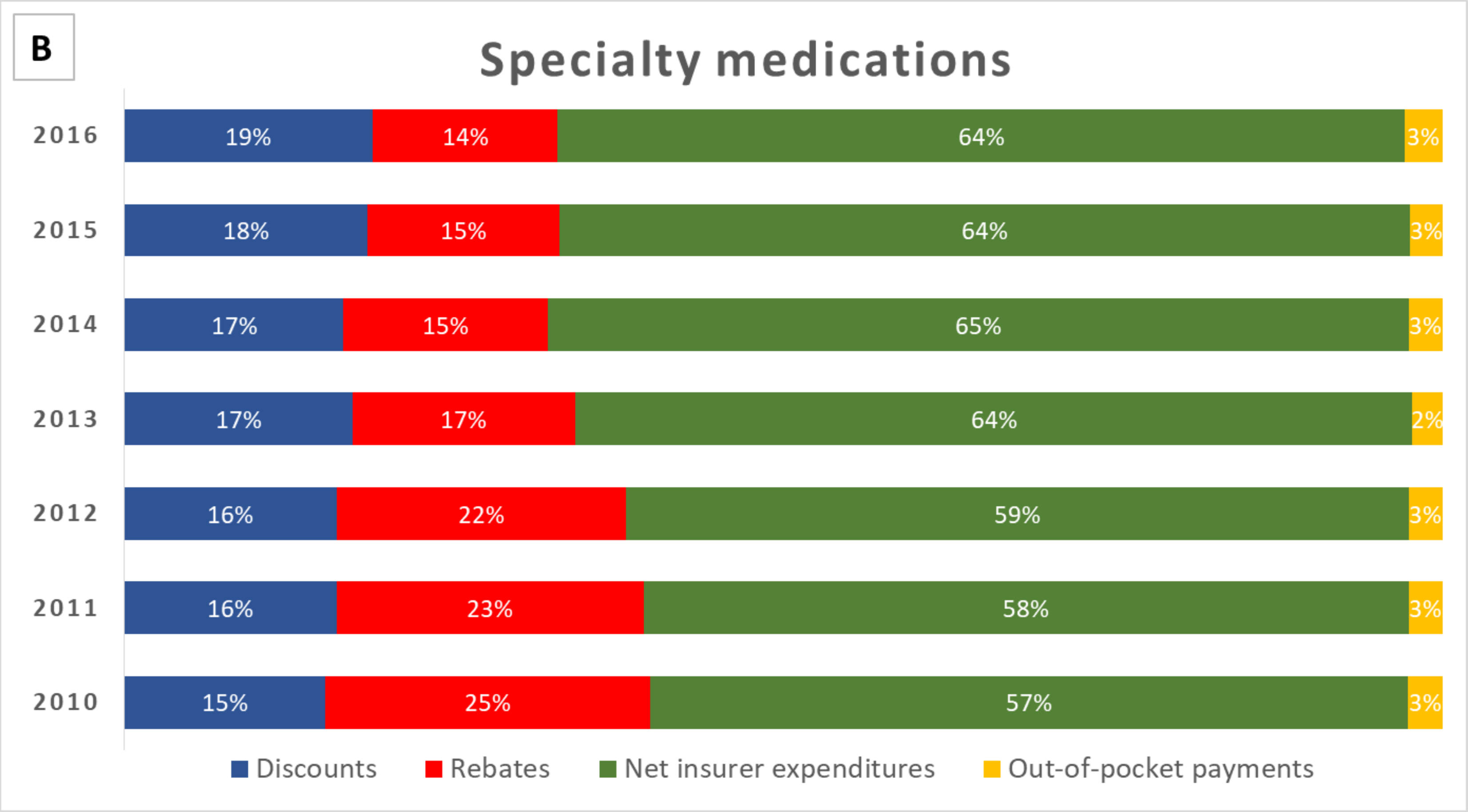

©2020 Yang EJ et al. *JAMA Network Open*

eTable 2. Median annual percent increase in list prices, price reductions, and payments for medications maintaining patent protection during each of the studied time periods. All results are represented as median annual percent increase (interquartile range). All results were adjusted to 2016 dollars using the Consumer Price Index (CPI)

	**2010-2014 (14 drugs)**	**2010-2015 (11 drugs)**	**2010-2016 (9 drugs)**
**AWP**	12.3% (10.1%-14.4%)	14.9% (11.0%-16.1%)	14.8% (10.0%-15.1%)
**Discounts**	15.6% (11.8%-18.7%)	19.4% (15.6%-20.3%)	17.5% (14.9%-20.9%)
**Rebates**	5.0% (-16.3%-20.6%)	14.3% (-0.8%-28.3%)	12.6% (-3.3%-25.6%)
**Insurer payments**	12.3% (2.7%-16.6%)	8.1% (3.6%-14.6%)	8.5% (4.3%-14.1%)
**Patient payments**	6.1% (4.6%-8.0%)	7.6% (5.1%-9.0%)	7.3% (6.0%-10.5%)

AWP = Average Wholesale Price

©2020 Yang EJ et al. *JAMA Network Open*
